# Feasibility and Safety of Continuous and Chronic Bilateral Deep Brain Stimulation of the Medial Forebrain Bundle in the Naïve Sprague-Dawley Rat

**DOI:** 10.1155/2015/256196

**Published:** 2015-04-15

**Authors:** Luciano L. Furlanetti, Máté D. Döbrössy, Iñigo A. Aranda, Volker A. Coenen

**Affiliations:** Laboratory of Stereotaxy and Interventional Neurosciences, Department of Stereotactic and Functional Neurosurgery, University Medical Center Freiburg, Breisacher Strasse 64, 79106 Freiburg, Germany

## Abstract

*Objective*. Deep brain stimulation (DBS) of the superolateral branch of the medial forebrain bundle (MFB) has provided rapid and dramatic reduction of depressive symptoms in a clinical trial. Early intracranial self-stimulation experiments of the MFB suggested detrimental side effects on the animals' health; therefore, the current study looked at the viability of chronic and continuous MFB-DBS in rodents, with particular attention given to welfare issues and identification of stimulated pathways.* Methods*. Sprague-Dawley female rats were submitted to stereotactic microelectrode implantation into the MFB. Chronic continuous DBS was applied for 3–6 weeks. Welfare monitoring and behavior changes were assessed. Postmortem histological analysis of *c-fos* protein expression was carried out.* Results*. MFB-DBS resulted in mild and temporary weight loss in the animals, which was regained even with continuing stimulation. MFB-DBS led to increased and long-lasting *c-fos* expression in target regions of the mesolimbic/mesocortical system.* Conclusions*. Bilateral continuous chronic MFB-DBS is feasible, safe, and without impact on the rodent's health. MFB-DBS results in temporary increase in exploration, which could explain the initial weight loss, and does not produce any apparent behavioral abnormalities. This platform represents a powerful tool for further preclinical investigation of the MFB stimulation in the treatment of depression.

## 1. Introduction

Major depressive disorder (MDD) is a debilitating predicament with negative consequences on both the affected individual and those close to the suffering person. Today, the best therapies include psychotherapy, electroconvulsive, and drug treatment ones, together producing remission in 70–90% of sufferers; however, 10–30% of the patients remain resistant to all currently available treatment combinations. Deep brain stimulation (DBS), considered in the past uniquely in the context of motor symptoms observed in essential tremor, Parkinson's disease, and dystonia, has since 2005 also been tested in MDD [1–4]. The limited number of clinical studies targeting treatment-resistant MDD by DBS ranges from a case study [5] to small trials [6–9] and suggests that the approach both is safe and can show long-lasting efficacy in a significant number of patients who have not responded positively to other types of interventions. The stimulation targets selected in these clinical studies included the subcallosal cingulate, nucleus accumbens (NAC), and the habenula. The diversity of targets reflects the characteristics of the network-model of depression that states that aspects of the syndrome (e.g., cognitive, vegetative, and somatic) can arise from dysregulation of neuronal activity at numerous loci on the limbic-cortical circuitry [7, 10].

A recent clinical trial in treatment-resistant MDD patients stimulated bilaterally the superolateral branch of the medial forebrain bundle (slMFB) [9, 11]: a brain structure upstream from all previous targeted areas, and thereby potentially affecting their neural activity. The stimulation of these neuronal bundles showed stable and chronic antidepressive effects with rapid onset and a higher proportion of patients responding already at lower stimulation intensity than observed in previous studies [9]. The combination of effects suggests that the MFB could be an important future clinical target for neuromodulation in MDD.

The MFB is a key structure of the reward system. It connects (amongst others) the ventral tegmental area (VTA) bidirectionally with the nucleus accumbens (NAC) and the medial prefrontal cortex (mPFC). Typically, the MFB is regarded as a structure that is intimately related to drug abuse and overwhelming reward system activation [13]. Early experimental rodent research on electrical self-stimulation of the brain (ESB) apparently showed detrimental effects on the rodents' health inducing apparent “self-starvation” [14]. These studies had a very complicated and specific design in terms of food deprivation periods and were later repeated with similar results using other novelty stress signals than ESB of the MFB. Surprisingly, the results were similar [15, 16]. Although the results of the first experiments were later brought into a different perspective, the key message that endured over the years was the incompatibility of MFB stimulation with the welfare of the animal. Thus it is important to study the feasibility and the health implications of continuous MFB DBS in the rodent under more physiological conditions and to know whether further research of DBS of the human MFB is justified. The objective of the current study therefore was to establish chronic and continuous bilateral high frequency stimulation (HFS) of the MFB in rodents, with particular attention given to welfare issues, behavioral effects, postmortem validation of electrode placement, and identification of stimulated pathways. This is an essential first step in the eventual investigation of mechanisms of action and the neurobiological substrates of MFB neuromodulation. All animals received bilateral bipolar electrodes into the MFB at the level of the midbrain ascending dopaminergic projections and, with the exception of the implanted controls, received continuous and chronic stimulation lasting between 3 and 6 weeks. The short- and long-term consequences of the stimulation were analyzed via a combination of behavioral tasks and observations, food and liquid consumption and parameters of indirect metabolic activity, and histological assessment. The results suggest that bilateral MFB stimulation has a robust, reproducible, reversible but moderate impact on the animals' welfare as shown by the cycle of weight decrease, stabilization, return to pre-DBS weight, and the weight gain following the end of stimulation. MFB stimulation also resulted in neuronal activation in infralimbic and prelimbic cortices, in NAC, and the dorsolateral thalamus, as shown by* c-fos* expression. Furthermore, the data imply that the dynamics of the transient DBS effect could be dependent on age at the DBS onset. A transient increase in the explorative, searching, and SEEKING type of behavior was measured in the MFB stimulated animals consistent with the proposed role of this structure both in healthy individuals and in clinical depression [11, 17, 18]. Overall, the study demonstrated that the continuous and chronic bilateral stimulation of the MFB in rodents is not detrimental to the animal's health and can serve as a powerful platform to investigate the effects and mechanisms of neuromodulation in experimental models of depression.

## 2. Material and Methods

### 2.1. Animals Housing and Feeding

**Figure 1:**
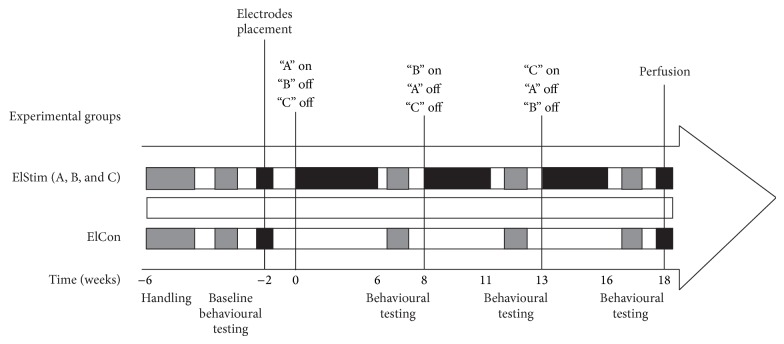
Experimental groups and study design.

Young adult female Sprague-Dawley (SD) rats (*n* = 16, Charles River, Germany), weighing 250 g, were housed in individual round cages (height: 40 cm; diameter: 40 cm), with the light/dark cycle maintained at 12 hours on and 12 hours off. Experimental groups and design are summarized in Figure 1. The study described in this paper had the approval of the Ethical Board of the University of Freiburg (*Regierungspraesidium;* TVA G10-124) and was carried out in accordance with the EU Directive 2010/63/EU concerning the protection of animals used for scientific purposes.

### 2.2. Surgical Procedures

Rats underwent general anesthesia induced and maintained by inhalation of isoflurane 2%. Skin disinfection was performed and followed by bilaterally stereotactic surgical implantation of custom-made bipolar electrodes into the MFB. Anterior-posterior (AP) and mediolateral (ML) coordinates are taken from bregma, dorsoventral (DV) coordinate from dura. AP = −4.4, ML = ±1.2, and DV = −7.8 [12]. The bipolar probes (125 *µ*m diameter each, 90% platinum/10% iridium, 15 mm long Teflon-coated shaft, World Precision Instruments, Sarasota, USA) were connected to a current isolator and fixed to the skull surface with microscrews and bone cement. Buprenorphine (75 *μ*g/Kg, i.p.) was given to all animals for postoperative analgesia.

### 2.3. Deep Brain Stimulation

Minimum of 12 days of recovery followed surgery before animals were connected to the pulse generator (STG 2008, Multichannel Systems, Germany) for stimulation. Stimulus pulses consisted of square-wave biphasic, constant current pulse pairs, and stimulation started using clinical relevant parameters (frequency 130 Hz, pulse width 100 *μ*s). Current was individually titrated for each cerebral hemisphere, beginning with 50 *μ*A and increased in units of 50 to a maximum of 350 *μ*A. The final current (mean 288 ± 8 *µ*A) was set 50 *µ*A less than the level that provoked side effects (e.g., rotation, hyperactivity). Safety measures were implemented during the experiment by regularly monitoring voltage and impedance (Hameg Instruments GmbH, Germany) in order to ensure the proper working of the system and to prevent excessive charge that could damage the brain-electrode interface [19, 20]. Based on weight and baseline behavioral data, the 16 animals were split into batches (“DBS A,” “DBS B,” “DBS C,” and control; *n* = 4 in all cases). At any one time chronic continuous MFB-HFS was applied to animals for 3 to 6 weeks. The control group had implanted electrodes but did not receive HFS.

### 2.4. Monitoring Health Status

In earlier investigations, the animals' welfare following MFB stimulation raised serious issues [14–16]. In the current study, rats were weighed twice a week and food and water were available* ad libitum*. Food consumed and feces produced were also assessed and used as indicators of metabolic activity [21].

### 2.5. Behavioral Assessment

#### 2.5.1. Home Cage Locomotor Activity

Exploratory behavior was evaluated in the home cage at different time points. Animals were filmed for 6 hours (1200–1800)/day for 5 days, with the first day of filming happening prior to turning on the stimulator, and the following days with the stimulator being on; similarly, filming started on the last day the stimulator was on and continued for 4-5 days afterwards. The bottom of the cage was virtually split on the screen into four equal quadrants, and complete line crosses from one quadrant to another area were counted. A blinded assessor scored the recordings.

#### 2.5.2. Forced Swim Test (FST)

FTS is used preclinically to evaluate and screen antidepressant effect of drugs or other treatments in rodents [22, 23]. The protocol consists in placing the animal into a cylindrical receptacle (40 cm height, 20 cm diameter) filled with water (25°C) and measuring the time of activity and immobility. The water level was adjusted so that the rat could not touch the bottom of the container with its tail and could not escape from the cylinder either. Behavior activity was recorded for 5 minutes by a digital video camera connected to the workstation (Viewer^2^, Biobserve, Germany). The amount of time spent in a posture of immobility was calculated. FST was performed before and after MFB-DBS.

#### 2.5.3. Elevated Plus Maze

Anxiety behavior was assessed by the elevated plus maze test, performed both prior to and after MFB stimulation, according to previous protocol [24]. The animals were placed in the center of the maze, consisting of two open arms and two enclosed arms. The amount of time spent in the open arms (in percent) and the number of entries into the open arms over 5 minutes were assessed (Viewer^2^, Biobserve, Germany).

#### 2.5.4. Quantifying Sucrose and Water Consumption

Water and 10% sucrose drinking behavior in the home cages was assessed separately at regular intervals over 24-hour observation period including both before and after stimulation.

### 2.6. Immunohistochemistry and Histological Analysis

Twelve weeks after surgery, animals were terminally anesthetized by an overdose of 10% ketamine (Bela-Pharm GmbH & Co. KG, Germany) and 2% xylazine (Rompun, Bayer-Leverkusen, Germany) and intracardially perfused with a solution containing 4% paraformaldehyde and 0.025% glutaraldehyde in 0.1 M phosphate buffer at pH 7.4. The brains were removed from the skull, kept in 30% sucrose at 4°C until they sunk, and cut into 40 *μ*m coronal sections. The free-floating sections were incubated with 1.5% H_2_O_2_, 1% sodium borohydride, and 1% milk powder, each in 0.02 M sodium phosphate buffer at pH 7.4 for 30 min, and exposed to a primary antibody raised in goat against* c-fos* (SC-52-G, 1 : 2000, lot. number K1808/F1109/A2810, Santa Cruz Biotechnology Inc., Santa Cruz, USA), or mouse anti-ED1 (1 : 1000 #MAB1435, Merck Millipore, KGaA, Darmstadt, Germany). After incubation for 48 h at 4°C, visualization of antibody-binding sites was based on DAB staining using biotinylated anti-goat (BA-5000; 1 : 200; Vector Laboratories, Inc., Burlingame, USA) or biotinylated anti-mouse (BA-2001; 1 : 200; Vector Laboratories, Inc., Burlingame, USA) as secondary antibody and avidin-biotin-technique (ABC Elite; Vector Laboratories, Burlingame, CA) for signal intensification. Finally 3,3′-diaminobenzidine (DAB; Merk, Darmstadt, Germany) and 0.01% H_2_O_2_ were used to develop the color reaction. After ED-1-DAB staining, sections were double-stained for Hematoxylin & Eosin (H&E), using a standard protocol. The sections were mounted on super frost plus slides (Langenbrinck, Emmendingen, Germany), dehydrated in ascending alcohol solutions, and cleared in xylene before they were cover slipped with Histofluid (Marienfeld Laborglas, Lauda-Königshofen, Germany) [25].

Assessment of the final electrode position was carried out by overlapping the H&E histological sections and the respective slice found on a standard stereotaxic rat brain atlas. The final coordinates were then plotted in a schematic figure for optimal visualization of the targeted area. The areas and networks affected by the MFB-HFS were assessed semiquantitatively by microscopically examining (Cell P, Olympus Soft Imaging Solutions, GmbH, Münster, Germany) the pattern of* c-fos* expression across different regions (Nucleus Accumbens, Prelimbic Cortex, mediodorsal thalamic nuclei and lateral habenula, somatosensory cortex, and dorsolateral striatum).* Fos* immunolabeling of cellular nucleus in target brain regions was interpreted as an indicator of neuronal activation induced by stimulation of the MFB [26–28].

### 2.7. Statistics

Two- or three-way ANOVAs with repeated measures was used (Statistica, Germany). In all cases, the main effects were tested for groups (DBS A, DBS B, DBS C, and control) and time (days or periods of DBS or before/after DBS); when analyzing the EPM data, the main effect of arm (open, closed) was also used. When appropriate, post hoc analyses were performed using Student-Newman-Keuls test. Level of significance was set at *P* < 0.05. Results are expressed as means ± standard error of the mean (SEM).

## 3. Results

### 3.1. Analysis of Health Status

**Figure 2:**
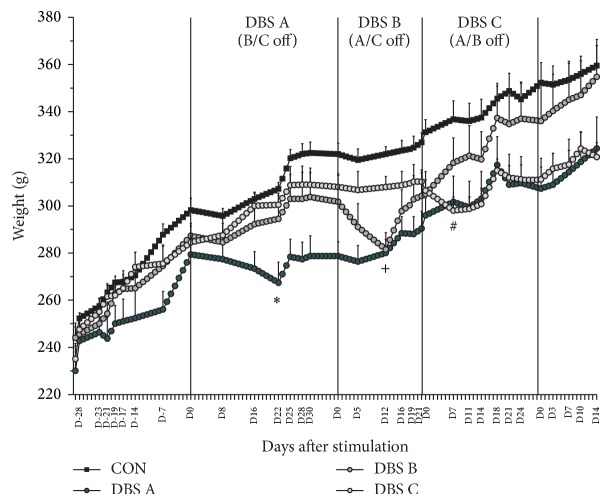
Weight dynamics. The animals' weight was closely monitored throughout the study and interpreted as a reflection of their health and vigor. The onset of continuous stimulation resulted in a temporary reduction by about 4–6% of the animals' weight compared to the prestimulation value. However, the number of days to reach the lowest and to recover to prestimulation weight, with the ongoing stimulation, differed across DBS A, B, and C. The figure shows the mean weight ± S.E.M. on the days when measurement was taken (labelled on the *x*-axis). The other values are estimates to show the evolution of the weight. See text for more details. ^∗^ *P* < 0.05 DBS A compared to all other groups; ^+^ *P* < 0.05 DBS B compared to C and controls; ^#^ *P* < 0.05 DBS C compared to controls.

During the study animals were weighed regularly; water and 10% sucrose consumption, food intake, and digestion (via the collection of feces) were monitored. Overall, the treatment did not have a significant bearing on the animals' weight dynamics (Figure 2; group, *F*(3,12) = 3.43; n.s.). However, MFB-HFS had a robust, but mild and temporary reduction in weight immediately following the onset of the stimulation in all animals (Group × Time, *F*(78,229) = 3.52; *P* < 0.001). This phenomenon was manifested as a provisional dip in the growth curve of the animals; following a variable period, despite the continuous stimulation the animals continued to gain weight normally. The relative weight loss was identical (4–6% of prestimulation weight), but the dynamics of it differed across the batches in terms of (i) the number of stimulation days until lowest weight measured and (ii) the number of stimulation days until regaining pre-DBS weight. The data suggests that the effects of the stimulation on the observed parameters are age/weight dependent. DBS A subjects were the youngest (13 weeks) and the lightest (279 ± 9 g) when stimulation started, with the slowest rate of weight loss (maximum after 22 days of MFB-HFS) and the longest recovery time of prestimulation weight (25 days). Following the onset of stimulation, DBS-B (19 weeks/302 ± 11 g) lost weight over 12 days, but recovered prestimulation weight after 19 days. Finally, DBS C rats were the oldest and heaviest (23 weeks/307 ± 5 g) with the quickest rate of reaching the lowest weight (after 7 days) and the fastest recovery of prestimulation weight (16 days). In all three cases, the day when the stimulated animals reached their lowest weight (DBS A, D22; DBS-B, D12; DBS-C, D7), the weight was significantly lower compared to the groups who had not been stimulated yet (Group × Time, *F*(78,229) = 3.52, *P* < 0.001). The electrode implanted/unstimulated control animals showed continuous weight gain throughout the study. The heterogeneity in the rate and duration of the weight loss might be related to the age and maturation of the CNS of the rats at the time of stimulation, but this needs further experimental evidence.

**Figure 3:**
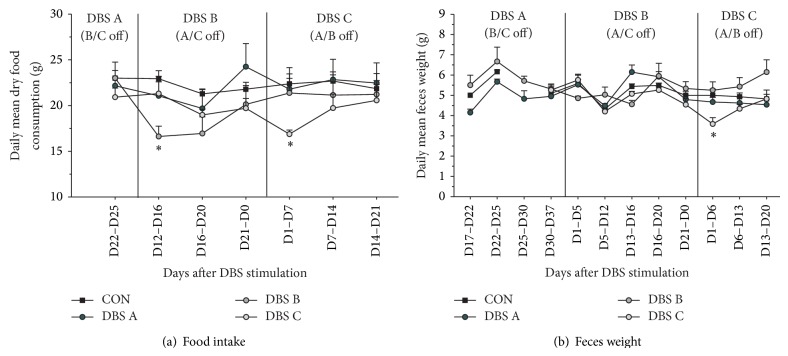
Food intake and metabolic rate. The animals' average food consumption and feces production were periodically measured to follow the impact of chronic continuous MFB-DBS. Following the onset and up to 16 days after stimulation, MFB-HFS animals consumed approximately 25% less food pellets compared to the unstimulated animals. However, beyond this initial period, the food intake recovered to normal levels even with continuous stimulation (a). Average feces production over time was stable, but an approximate reduction of 25% was observed during the first week of MFB-HFS in the DBS C group. See text for more detail. ^∗^ *P* < 0.05 compared to all other groups during the same period.

**Figure 4:**
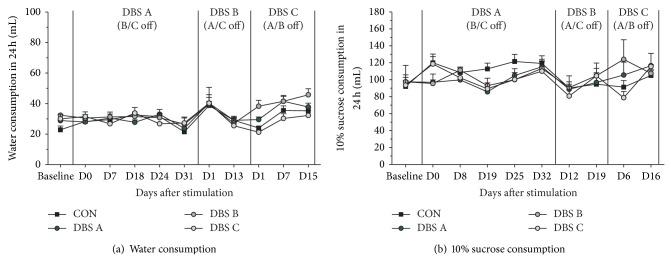
Liquid consumption. As in indicator of welfare, liquid consumption over a 24-hour period was periodically monitored during the study. The animals' consumption of neither water (a) nor 10% sucrose (b) was influenced by MFB-HFS during the study period.

There was no overall difference across the groups in their average daily dry food consumption (Figure 3(a), Group *F*(3,12) = 0.9, n.s.), but when analyzed over time, animals receiving MFB-HFS reduced their intake by about 25% for up to 20 days (Group × Time, *F*(18,72) = 2.93, *P* < 0.001), which also explains the temporary stimulation induced weight loss. Similarly, overall across the study there were no group differences in feces production (Figure 3(b), group, *F*(3,12) = 1.79, n.s.). However, up to 6 days of stimulation, DBS B showed a tendency and DBS C a significant reduction of about 25% in feces production (Group × Time, *F*(24,96) = 3.08, *P* < 0.001). Importantly when considering welfare issues MFB-HFS did not have an impact on either water consumption (Figure 4(a), Group × Time *F*(10,140) = 0.75, n.s.) or 10% sucrose consumption throughout the period of the study nor during the stimulation phases (Figure 4(b), Group × Time *F*(9,126) = 1.40, n.s.).

### 3.2. Behavioral Assessment

**Figure 5:**
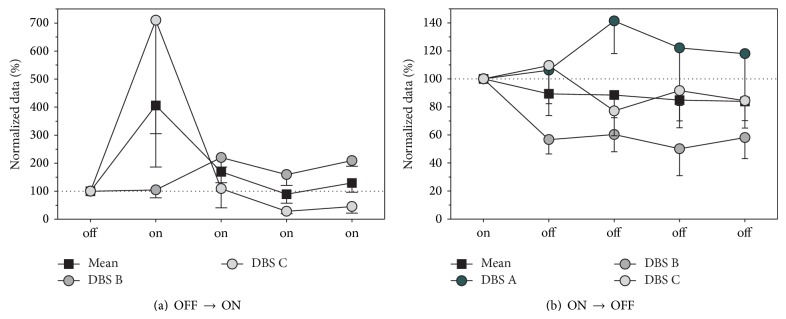
Home cage activity (HCA). Changes in the locomotor activity, reflecting explorative and searching behavior, were assessed in the animals' home cage prior to MFB-HFS and during the first 4 days of stimulation (a). During the early period of MFB-HFS, on average, most animals tended to increase their locomotor activity for at least 72 hours, some for longer periods. At the end of the stimulation period changes in locomotor activity coinciding with the turning-off of the stimulation were measured by using the final day of stimulation as the baseline and comparing it to locomotor activity over the first 4 days without stimulation (b). On average, switching off the MFB-HFS induced a reduction in HCA. See text for more detail.

Home cage locomotor activity (HCA) was assessed (i) on the day prior to MFB-HFS and the first 4 days of stimulation and (ii) on the final day of MFB-HFS and the subsequent 4 days with the stimulator off. HCA showed an overall mean increase across animals of 406 ± 220% and 169 ± 39% during the first and second day of stimulation, respectively, compared to the normalized activity levels prior to stimulation. On the final day of activity monitoring, average levels were still above the baseline level (130 ± 33%); however there was variation across batches (Figure 5(a), Group × Day, *F*(4,24) = 3.27, *P* < 0.05). When the HFS was turned off, on average, the opposite trend was observed: compared to the final day of stimulation, the activity levels decreased to between 89 ± 16% and 84 ± 15% during the following 4 days (Figure 5(b)). However, the overall stimulation duration could have an impact on the HCA measurement after stimulation as DBS B with the shortest stimulation (21 days) manifested the most reduction in activity compared to DBS C (27 days) and DBS A (38 days).

**Figure 6:**
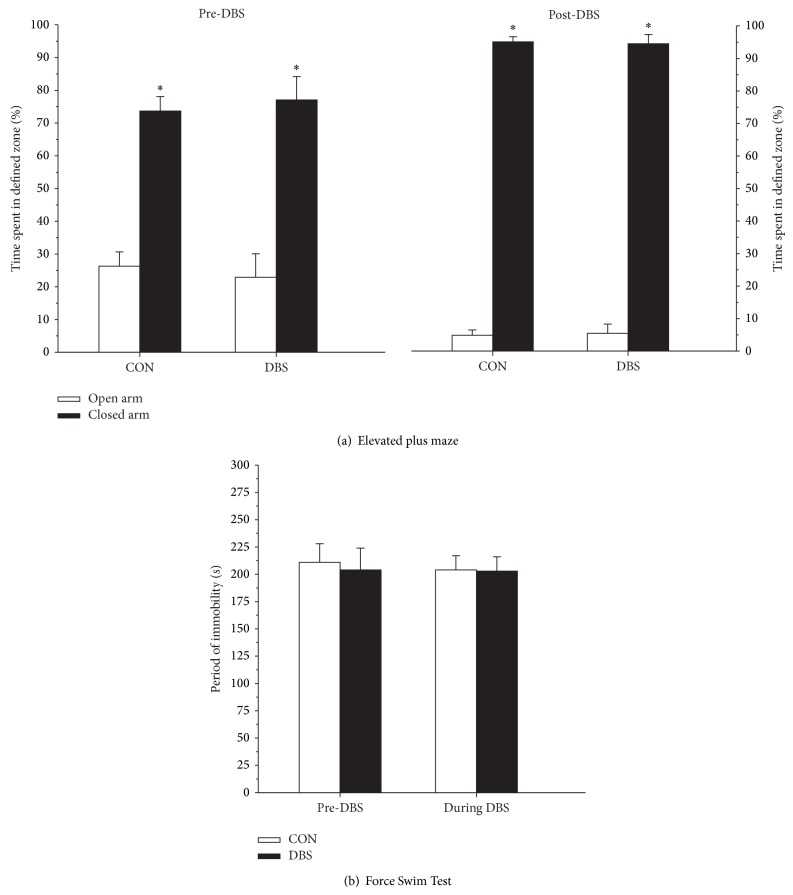
Behavioral testing. MFB-HFS did not have any specific effects on the animals' anxiolytic behavior as measured by the elevated plus maze: stimulated animals performed identical to the controls both before and after stimulation (a). Similarly, the stimulation had no impact on the animals' performance in the Forced Swim Test, a measure of behavioral despair (b). See text for more detail. ^∗^ *P* < 0.05 closed arm compared to open arm.

Tests confirmed that the chronic MFB-HFS did not induce any behavioral abnormalities in the animals. Changes in the anxiolytic behavior were measured using the elevated plus maze before and after stimulation in the control and experimental animals. The data shows that all tested animals spent similar percentage of the time in the “closed arm” and that this proportion increased in a similar fashion across the groups between the two testing sessions (Figure 6(a), Group × Session *F*(1,12) = 0.72, n.s.). Similarly, performance on the Forced Swim Test, measuring behavioral despair, was not affected by chronic and continuous MFB-HFS (Figure 6(b), Group × Session, *F*(1,13) = 0.18, n.s.).

### 3.3. Histology

**Figure 7:**
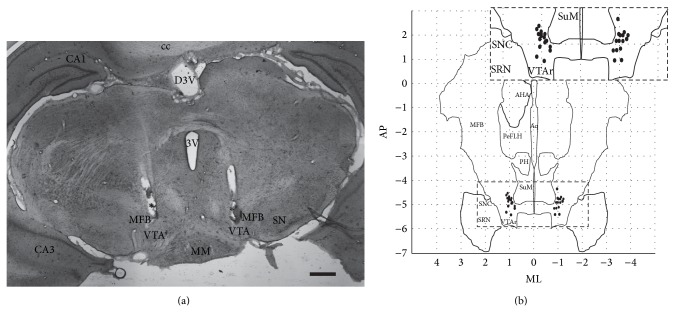
Histological assessment. Electrode placement was assessed using H&E staining of brain slices (a), with the bilateral positions of individual electrode tips summarized (b). Electrode tips were within the dorsoventral range of −8.0 ± 0.1 mm, according to Paxinos Rat Brain Atlas [12]. CA1: CA1 field of the hippocampus; CA3: CA3 field of the hippocampus; MPA: medial preoptic area; mfb: medial forebrain bundle; PeFLH: perifornical part of lateral hypothalamus; AHA: anterior hypothalamic area; PH: posterior hypothalamus; 3V (dorsal): third ventricle; MM: medial mammillary nucleus; cc: corpus callosum; SuM, supramammillary nucleus; VTA: ventral tegmental area; SNC: substantia nigra pars compacta; SRN: substantia nigra pars reticulate; AP: anterior-posterior; ML: mediolateral. Scale bar = 500 *µ*m.

The H&E staining confirmed the consistency and accuracy of the bilateral electrode placements (Figures 7(a) and 7(b)). On the right, the mean electrode position was at AP: −4.9 ± 0.10 mm, ML: 0.9 ± 0.05 mm, and DV: −8.2 ± 0.1 mm and on the left side AP: −4.9 ± 0.1 mm, ML: 0.9 ± 0.05 mm, and DV: −8.0 ± 0.10 mm. Two out of 40 electrodes were misplaced or could not be identified in the histological sections (5%). The placement had a mean variation of 0.4 mm in the AP axis compared to the theoretical target; however, this still corresponds to the ascending dopaminergic mesolimbic projections.

**Figure 8:**
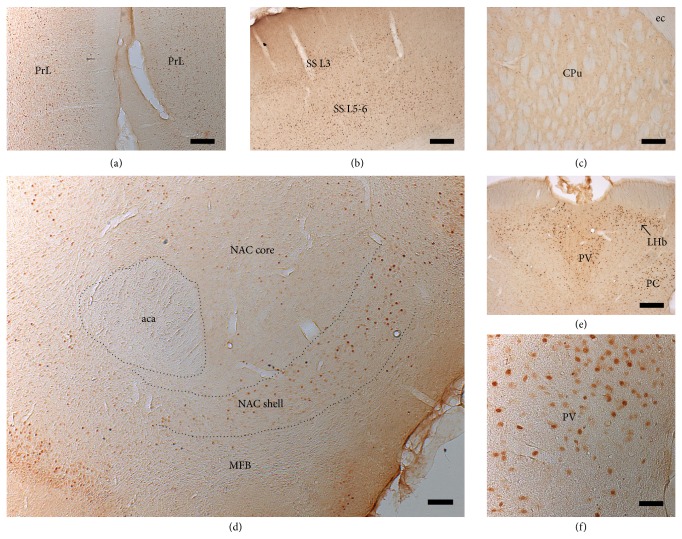
Immunohistochemical visualization of* c-fos* expression. Following MFB-HFS, strong* c-fos* expression was observed in prelimbic cortex (a), layers 3 and 5/6 of the somatosensory cortex (b), paraventricular thalamus, lateral habenula (e, f), and shell of the nucleus accumbens (d), suggesting that the stimulation activated mesolimbic and mesocortical pathways and its projection targets. However, little or no* c-fos* expression was observed in the dorsal striatum where the stimulated pathways do not project to. See Table 1 and text for more details. PrL: prelimbic cortex; NAC: nucleus accumbens; PV: paraventricular thalamic nucleus; LHb: lateral habenular nucleus; PC: paracentral thalamic nucleus; aca: anterior commissure; SS: somatosensory cortex; ec: external capsule; CPu: striatum. Scale bar = 50 *µ*m in (a), (b), (d), and (e); 250 *µ*m in (c) and (f).

Selective stimulation of the ascending ventral mesencephalic DA fibers, that is, originating from the VTA (A10), led to upregulation and increase of* c-fos* expression in the shell of NAC, prelimbic cortex, mediodorsal thalamic nucleus, and lateral habenula. The semiquantitative assessment of* c-fos* expression in these terminal structures of the dopaminergic MFB projections revealed a long-lasting upregulation following chronic continuous bilateral MFB-HFS (Table 1). The stimulation induced long-term plastic changes in neural activity as the upregulation was present even in the group DBS A for whom the stimulation seized for up to two months prior to perfusion. Conversely, the dorsolateral striatum that receives dopaminergic neurons mainly from the substantia nigra (A9) [29] did not present positive staining for this marker suggesting that predominantly the mesolimbic/cortical pathway was stimulated. A subset of animals also received an acute, 2-hour continuous stimulation just prior to perfusion, which resulted in strong upregulation of* c-fos*, particularly in the prelimbic frontal cortex and shell of the NAC (Figures 8(a)–8(f)).

## 4. Discussion

Acute electrical stimulation targeting the medial forebrain bundle (MFB) and associated pathways have been investigated in preclinical models of psychiatric disorders [30–33]; however, the consequences of chronic and continuous MFB stimulation have not been previously studied. The current experiment focused on welfare issues concerning bilateral chronic continuous high frequency stimulation (HFS) of the MFB in rats. The results confirm that clinically relevant stimulation parameters and conditions are feasible and safe and do not impose any apparent long-term welfare issues or behavioral changes in normal, nonpathological experimental animals. MFB-HFS induced acute neural activation resulting in the upregulation of* c-fos* expression in ascending target areas such as in the shell of NAC, infralimbic and prelimbic cortex, mediodorsal thalamic nucleus, and lateral habenula. Neural activation was observed even at two months following the end of stimulation suggesting that the MFB-HFS can induce long-lasting neural adaptation in pathway activity.

### 4.1. The MFB in Affective Neuroscience

The rodent MFB stretches from the ventral tegmental area (VTA) in the midbrain to several forebrain structures and includes both myelinated and short typically unmyelinated ascending and descending projections. Studies describe up to 50 fiber subcomponents and 13 neurotransmitters associated with the MFB [34–37], and novel regulatory elements of the MFB are still being discovered [38]. The MFB hosts the mesolimbic and the mesocortical fibers connecting the midbrain dopaminergic (DA) neurons of the VTA to the NAC and the prefrontal cortex, subserving reward, motivational, and learning pathways, and associated with the neural circuitry of psychiatric disorders such as addiction and depression [29, 39]. Crucially, within the framework of affective neuroscience, the ascending DA projections are considered as the neural substrate for the SEEKING system, one of several hard-wired primary affective systems, which ensures positive emotional and euphoric behaviors supporting exploration and modifies appetitive learning [17, 18, 40–42]. Key symptoms in clinical depression and in experimental models, such as anhedonia and helplessness, are thought to be the result of the hypoactivity of the SEEKING system, and the modulation of this system is considered as a potential therapeutic strategy [18, 42].

### 4.2. Feasibility and Safety of Bilateral Continuous and Chronic MFB-HFS

Early experiments by Olds and Milner and later by many others using intracranial self-stimulation (ICSS) of the MFB set the ground for research into the neurobiology of addiction, reward, and motivation [43–45]. However, historical data suggested that long-term and continuous stimulation of the MFB is not viable as it is detrimental to the animals' health. A series of studies showed rats implanted with MFB electrodes, when given the choice between self-stimulation and food, would choose self-stimulation over food even when this leads to starvation. The prevailing idea over the last four decades was that the rewarding effect of MFB stimulation trumps the need to eat [14–16].

The current results unambiguously refute this preconceived idea. Animals with electrodes implanted bilaterally into MFB, external to the VTA, but affecting the transmission of ascending midbrain A10 dopaminergic neurons, received continuous clinically relevant HFS up to 6 weeks. Although the stimulation had a reproducible impact on the animals' weight, food intake, and metabolism, changes in these parameters were temporary and affected mildly the animals' welfare. The weight decrease, observed immediately with onset of MFB-HFS, stabilized within 1–3 weeks and was followed by sustained weight gain despite the ongoing, chronic electrical stimulation. The start of MFB stimulation also coincided with an temporary (observed up to 72 hours) increase in the explorative, searching, and SEEKING type of behavior, which is consistent with the function of the MFB [17, 40] (see Video 1 in Supplementary Material available online at http://dx.doi.org/10.1155/2015/256196). However, even during the period of weight loss, the chronic and continuous MFB-HFS did not affect the animals' water or sucrose consumption suggesting that the animals continued to have and could meet their physiological needs. Furthermore, the chronic MFB-DBS did not produce any apparent functional impairment as measured by tests of anxiety or behavioral despair in the normal, nonpathological experimental animals used.

There are several reasons why the current results contrast with the historical data. Since Olds and Milner, electrical stimulation in preclinical studies has been synonymous with ICSS, and it is only during the last 10–15 years that DBS has been adapted to preclinical studies. Routtenberg's works are convincing within the context they were performed in, but there are crucial differences between “rat-led” ICSS and “investigator-led” DBS experimental designs. Firstly, in ICSS there is the crucial conditioned association between an action and the subsequent reward sensation, which urges the animals to persevere with the lever pressing. In the current study, the MFB stimulation was chronic and this was not conducive for an association to be constructed between the rat's action and the sensation produced by the HFS. Secondly, in the historical studies animals were given the choice between food and the more salient ICSS of the reward pathway. In the current study, animals had no options but had* ad libitum* food access. ICSS has proved to be an excellent tool to study addiction and reward pathways [31, 32]. However, between the two types of preclinical electrical stimulation, DBS is more refined and adapts to altering pathological behaviors or modulating deregulated neural pathways.

### 4.3. Multiple Target Activation following MFB-HFS

Over the years the stimulations of numerous regions associated with the neurocircuitry of depression have been targeted in depressed patients. Stimulations of the subgenual cingulate cortex (Cg25), the ventral capsule/ventral striatum, or the NAC have shown variable response and remission rates [1, 6, 46, 47]. Following the superolateral MFB stimulation, robust antidepressant efficacy in patients was observed within days as opposed to a longer timescale seen in other studies [9, 48]. The reason for the near-immediate effect is not yet known; however, it could be due to the position and the capacity of the MFB to modulate/synchronize the function of all the other key structures implicated in the network. For example, MFB stimulation has been shown to increase the activity in mesolimbic and mesocortical dopamine pathways by enhancing descending glutamatergic excitatory afferents to the VTA and thereby increasing VTA cell firing [49–51]. Stimulation could also influence VTA activity, leading to symptom relief, by increasing lateral hypothalamic (LH) orexin release in the VTA [52–54] or via LH's GABAergic afferents in the VTA [55]. Additional preclinical studies in validated animal models of depression, for example, incorporating micro-PET or in vivo voltammetry methods, are needed to shed light on the neural basis of MFB-HFS's antidepressive effect [42].

Other investigators have shown that DBS induced neural network activation can be followed by looking at the expression of the protooncogene,* c-fos*, and subsequent synthesis of Fos proteins, via immunohistochemistry [26, 27]. The current study confirmed that MFB stimulation can modulate neuronal activity in multiple regions: following HFS, robust upregulation of* c-fos* was observed in the shell of the NAC, infralimbic and prelimbic cortex, somatosensory cortex, mediodorsal thalamic nucleus, and lateral habenula. Similar patterns of* c-fos* upregulation were described by others following short-term ICSS of the MFB [27, 28]. However, in addition to demonstrating short-term modulation, lasting 1-2 hours, the current study has also shown long-term adaptation in neural activity in multiple targets as increased* c-fos* activity was sustained for up to two months following the end of stimulation.

## 5. Conclusions

Recent clinical work targeting the MFB by DBS in treatment resistant depression confirmed the MFB as a promising target that should be investigated more preclinically. The current study demonstrated two critical points. Firstly, continuous and chronic bilateral stimulation of the MFB in rodents is safe and without any consequences on welfare: the approach can be integrated into future investigations of neuromodulation in validated experimental models of depression. Secondly, MFB stimulation results in neuronal activation of multiple regions implicated in the neurocircuitry of depression, such as prefrontal areas, including infralimbic and prelimbic cortices, the NAC, and the dorsolateral thalamus, as shown by upregulation of* c-fos* expression. This confirms the strategic position of the MFB as its stimulation recruits multiple afferent and efferent connections, inducing short- and long-term plastic adaptations in local and distal neuronal activity. The platform represents a powerful preclinical tool to investigate the role and the neurobiological substrates of the MFB and its associated pathways and targets in the treatment of depression.

## Supplementary Material

The video clip presents SEEKING behavior induced by chronic continuous bilateral bipolar stimulation of the medial forebrain bundle in a rat, during the titration phase of our stimulation protocol. The video starts showing the animal's expected behavior in OFF condition. During the first few seconds of stimulation ON, visual fixation and freezing behavior can be observed, followed immediately by intensive sniffing and subsequent persistent explorative behavior of the home-cage and surrounding environment. The anti-TH micrographs enclosed in the video indicate the positioning of the stimulating electrodes in the MFB. MFB: Medial Forebrain Bundle; NAC: Nucleus Accumbens; CPu: Striatum; SNc: Substantia Nigra pars compacta; VTA: Ventral Tegmental Area; RRF: Retrorubral field. Yellow spots on the micrographs schematically represent the electric field within the stimulated area.

## Figures and Tables

**Table 1:** Semiquantitative analysis of region specific early gene *c-fos* expression across the experimental animals.

Animal	Group	Prelimbic cortex	Nucleus accumbens shell	Somatosensory cortex	Dorsomedial thalamic nuclei	Dorsolateral striatum
1	Control	+	−	−	+	−
6	Control	+	−	+	+	−
9	Control^*^	++	++	++	++	−
13	Control^*^	+++	++	+++	+++	++
2	DBS A	++	++	+	++	+
4	DBS A	+	++	−	++	−
8	DBS A^*^	++	+	++	++	−
10	DBS A	++	+++	+++	++	−
3	DBS B	+	+	+	+	−
12	DBS B	+++	+++	+++	++	+
15	DBS B^*^	++	++	+	++	+
20	DBS B^*^	++	++	−	++	−
7	DBS C	+	+	+	+	−
11	DBS C	+++	+++	++	+++	−
17	DBS C	+++	+++	+++	+++	+
18	DBS C^*^	+++	+++	++	+++	−

– no expression; + mild; ++ moderate; +++ high expression; asterisks indicate a subgroup of animals stimulated for 2 hours preceding perfusion.
